# The theory of parallel climate realizations as a new framework for teleconnection analysis

**DOI:** 10.1038/srep44529

**Published:** 2017-03-23

**Authors:** Mátyás Herein, Gábor Drótos, Tímea Haszpra, János Márfy, Tamás Tél

**Affiliations:** 1 MTA–ELTE Theoretical Physics Research Group, Budapest, Hungary; 2 Institute for Theoretical Physics, Eötvös University, Budapest, Hungary

## Abstract

Teleconnections are striking features of the Earth climate system which appear as statistically correlated climate-related patterns between remote geographical regions of the globe. In a changing climate, however, the strength of teleconnections might change, and an appropriate characterization of these correlations and their change (more appropriate than detrending the time series) is lacking in the literature. Here we present a novel approach, based on the theory of snapshot attractors, corresponding in our context to studying parallel climate realizations. Imagining an ensemble of parallel Earth systems, instead of the single one observed (i.e., the real Earth), the ensemble, after some time, characterizes the appropriate probabilities of all options permitted by the climate dynamics, reflecting the internal variability of the climate. We claim that the relevant quantities for characterizing teleconnections in a changing climate are correlation coefficients taken over the temporally evolving ensemble in any time instant. As a particular example, we consider the teleconnections of the North Atlantic Oscillation (NAO). In a numerical climate model, we demonstrate that this approach provides the only statistically correct characterization, in contrast to commonly used temporal correlations evaluated along single detrended time series. The teleconnections of the NAO are found to survive the climate change, but their strength might be time-dependent.

Recent results^1^,^2^,^3^,^4^,^5^ indicate that the mathematical concept that provides an appropriate framework for the description of the climate’s internal variability is that of snapshot^6^,^7^ or pullback attractors^1^,^8^,^9^. These are sets in the high-dimensional phase space reached with exponential accuracy after a finite transient time. Most importantly, these sets carry a unique probability distribution, reflecting the internal variability, which is an intrinsic characteristic of the climate system. This property follows from the general feature of dissipative systems that they forget their initial conditions, hence after the transient time the probability distribution is independent of how the system was prepared. In numerical modeling, this probability distribution is faithfully represented by an ensemble of simulations. The members differ only in their numerical initial conditions, and their behaviour is characteristic for the climate system after the transient time. The difference between the members, i.e., the ensemble spread cannot be reduced, since it just represents the internal variability.

We emphasize that this spread does *not* represent some uncertainty of a climate projection, instead, provides to the projection a probabilistic nature which defines *correctly* the climatological statistics of the physical, e.g. atmospheric, quantities. Note that, in contrast to ensemble weather forecasts, the projection time here is long enough, on the order of a few decades, for the ensemble members to forget their initial conditions. We note that an ensemble approach has become well-known in assessing the impact of internal variability on short-term climate-related predictions, up to the time scale of a decade (see e.g. refs 10, 11, 12, 13, 14). Such works evaluate ensemble means and standard deviations on the scale of the predictability time of the problem in question, i.e., while the distribution of the ensemble members *do* depend on initial conditions. Their spirit is thus similar to that of ensemble weather forecasts, and the used ensembles cannot represent snapshot attractors and the corresponding probability distributions. The distinctive property of our approach is that it is valid after a convergence to the probability distribution on the attractor took place.

Qualitatively, the members of an ensemble representing the snapshot attractor in a climate model can be viewed as *parallel climate realizations* evolving on different Earths subjected to the *same* set of physical laws, boundary conditions and external forcing. In what follows, we shall use the term “parallel climate realizations” for representing the probability distribution on the snapshot attractor, i.e., after the aforementioned transient time has passed which turns out to be on the order of a few decades. For more details on snapshot attractors and their distributions see section The snapshot approach and refs 7 and 15. Thus our approach relies on a concept that is both physically and mathematically well-founded, which leads us define the climate as the union of all the members of the ensemble of the parallel climate realizations. We emphasize that this way we define a climate for each time instant. Note that these considerations also imply that any statistics is obtained correctly only if it is evaluated with respect to the plethora of the parallel climate realizations, and this is why we consider any results deviating from those obtained by the snapshot approach inappropriate.

When dealing with climate changes induced by shifting parameters (e.g. increasing CO_2_ concentration), the probability distribution and the ensemble statistics unavoidably change in time^7^. The viability of the snapshot approach is clearly demonstrated^15^ in the framework of an intermediate-complexity climate model, the Planet Simulator (PlaSim)^16^, by ensuring the ensemble to converge to the snapshot attractor.

Teleconnections are recognized as statistical correlations in climate-related patterns between remote geographical regions of the globe. These correlations are commonly defined involving so-called indices (e.g. those of ENSO [El Niño–Southern Oscillation], PNA [Pacific–North American pattern] and NAO [North Atlantic Oscillation]) which are based on observational data, and these teleconnections are intensely studied (see e.g. refs 17, 18, 19, 20, 21, 22, 23) since they may have essential impact on weather patterns^24^.

Teleconnections are special structures within the internal variability, which naturally calls for the picture of the parallel climate realizations. In our paper we show that the statistics of teleconnections can only be characterized properly via this approach. The challenge is that teleconnections are often defined using correlation coefficients evaluated with respect to time over certain time windows^25^,^26^,^27^ without taking into account the effects of externally induced trends in the relevant time series which may, however, play an important role^28^,^29^,^30^,^31^. More careful definitions apply detrending to the time series^23^,^29^,^30^,^32^,^33^. In both of these methodologies correlation coefficients are evaluated over *single time series*. The problem with using time windows is that they are ambiguous in a changing climate as we illustrate in our paper. We claim that, instead, correlation coefficients should be evaluated over the ensemble. This can be carried out in any instant of time, which opens the opportunity to study even the *time evolution* of correlation coefficients and hence that of teleconnection patterns (which is an aim appearing with increasing emphasis in the literature^30^,^32^,^34^,^35^). In a broader context, a basic feature of the approach of parallel climate realizations is the capability of a clear separation of externally induced trends from internal fluctuations (appearing in any individual climate realization). The former are obtained as ensemble averages. As a consequence, a “hockey stick” shaped signal (well known in observed global mean temperature time series) can only be considered a sign of a climate change if it also appears in the ensemble average. Furthermore, the statistics of the internal fluctuations should also be investigated in the ensemble framework proposed here.

Our results are intended to serve as a proof of concept for the proposed method. Therefore, we do not consider the details (geographic patterns, internal time scales etc.) of the NAO phenomenon, rather we focus on the simplest quantifiers of teleconnections: on correlation coefficients. For two chosen climatic variables, their correlation coefficient characterizes how synchronously their anomalies (the difference from the variable’s expectation value, divided by its standard deviation — also interpreted as fluctuations) appear from a probabilistic point of view. The question is how we can obtain the probabilistically correct correlation coefficients in a changing climate.

## Setup

**Figure 1:**
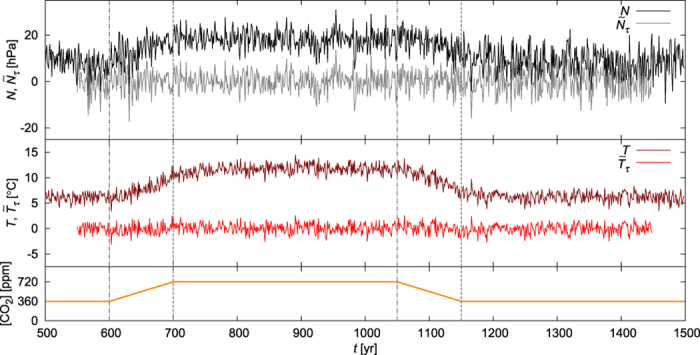
The NAO signal *N* of (1) and the corresponding DJF surface mean temperature *T* of the Mediterranean in PlaSim in one of the particular climate realizations (*i* = 16). The detrended signals 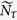 and 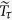 are also included: detrending has been performed by subtracting a moving average from the original signals, where the moving average is calculated over a time window of length *τ* = 101 yr. The forcing (i.e., the CO_2_ concentration) is also plotted as a function of time. The vertical dot-dashed (dashed) lines in grey mark the beginning (end) of the CO_2_ ramps.

To illustrate our argumentations, we consider NAO^25^,^36^,^37^ within a given climate change scenario in a standard Planet Simulator (PlaSim) setup (see section The Planet Simulator and the model scenario). The climate change is embedded into a 1500-year long CO_2_ scenario which contains a doubling of the concentration from 360 ppm over a century, starting in year 600, a symmetric decrease, and intervals of constant concentration before, between and after these ramps (see the orange graph of Fig. 1 and, for the precise formula, section The Planet Simulator and the model scenario). The ensemble of the parallel climate realizations is represented by an ensemble of 192 members, see also section The Planet Simulator and the model scenario.

A preliminary study^15^ illustrates that the NAO time series strongly differs in any individual climate realization from the ensemble average, and reliable climate trends can only be extracted from the latter. Here we evaluate correlation coefficients taken over the ensemble, and monitor their time evolution during a climate change. We emphasize, however, that this study is not intended to make any accurate projections for the Earth’s NAO phenomenon, rather to illustrate its fundamental variability, and the applicability of the concept of parallel climate realizations.

### NAO in a changing climate

The North Atlantic Oscillation, that is, the fluctuation of the difference of sea-level pressure between the Azores high and the Icelandic low, is a phenomenon which is observed to be in connection with climate-related patterns along the northern coasts of the Atlantic Ocean, i.e., in western Europe and in eastern North America. The phases of the NAO are described by the North Atlantic Oscillation index that has several possible definitions, all having in common that they try to capture these fluctuations in a particular season or month. In what follows, we shall consider the winter season as traditionally studied in the literature, see e.g. ref. 37.

It is commonly known that in the current Earth climate an above-than-average value of the NAO index typically implies a higher winter temperature in the Scandinavian region and in the eastern United States, and a lower temperature in the Mediterranean and Greenland^27^,^38^,^39^,^40^. Analogously, an above-than-average value of the NAO index is also related to more than the average precipitation during winters in Scandinavia, and less than the average precipitation over the Mediterranean.

In order to carry out a NAO teleconnection analysis in the PlaSim model, we define a simple PlaSim NAO signal. We pick two locations (grid cells) in PlaSim: one of them covers the Icelandic low (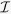), the other the Azores high (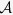). Our NAO signal is then simply the difference of the sea-level pressure 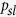 averaged over the winter season (months December, January and February, denoted as DJF afterwards) between the two grid cells:


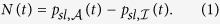


(See section The extraction of a NAO signal for further details.) We emphasize that, unlike in the common, “canonical” definition of the NAO index^25^, we do *not* take here the normalized sea-level pressures (obtained by subtracting the long-term mean and dividing by the long-term standard deviation, i.e., by calculating the anomaly with respect to the long-term behaviour). We demonstrate in Supplementary Figures S1–4 that thus there is no qualitative change when including the normalized sea-level pressures according to ref. 25, and illustrate thus that the method of parallel climate realizations can be applied with any definition for the relevant quantities. At the same time, defining a “long-term mean” and a “long-term standard deviation” is ambiguous in time series reflecting a changing climate, since the values of the physically relevant statistics are shifting in time^7^,^15^. For keeping the conceptual basis clear, we prefer not to manipulate the raw quantities 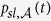 and 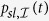 in (1) in the main text.

First we present, in Fig. 1, an example for what an observer could record in our climate change scenario for the NAO signal (1) and for the DJF surface mean temperature of the Mediterranean. It is obvious from Fig. 1 that trends, appearing as responses to the CO_2_ forcing, dominate the time evolution of the relevant quantities. In particular, both the NAO signal and the temperature increase and decrease during the increasing and the decreasing CO_2_ ramp, respectively. It would be erroneous to conclude from this that the common relation, a higher NAO signal implying a lower Mediterranean temperature, is broken.

Anyway, the task of the observer is to calculate, from these time series, some correlation coefficient that reflects faithfully the *probability distribution* that underlies the correlated fluctuations of these two quantities.

In Supplementary Discussion I we illustrate that the naive approach without somehow removing the trends might lead to dubious results. For the demonstrative purpose of our paper, we choose a simple but acknowledgedly efficient^41^ detrending scheme, namely, the subtraction of a moving average from the original time series. Supplementary Figure S5 illustrates that the correlation coefficients (analyzed in detail in the next section) do depend on the window length *τ* over which the moving average is taken, but this dependence turns out to be weak for 11 yr < *τ* < 101 yr. Therefore, restricting our investigation to one particular value of *τ* is reasonable in our problem. We take *τ* = 101 yr. In Fig. 1 we also illustrate the detrended version 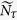 and 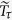, with *τ* = 101yr, of the original time series, over which we shall evaluate the correlation coefficients.

We note that it is usual to construct anomalies from such time series by subtracting the mean and dividing by the standard deviation, thus quantifying the above-than-average or below-than-average nature of the values in the time series. We skip, however, this traditional step here, because this is not needed for our argumentations.

### The ambiguity of the results based on single realizations

Once the detrended time series have been obtained, e.g. those in Fig. 1, one has to choose the time interval [*t*_1_, *t*_2_], of length Δ*t* = *t*_2_ − *t*_1_, over which the correlation coefficient should be evaluated.

One option is to calculate the correlation coefficient for a long time interval, encompassing most of the observation, e.g. [*t*_1_, *t*_2_] = [575 yr, 1425 yr] in our case. The correlation coefficient corresponding to the detrended time series of Fig. 1, calculated over this [*t*_1_, *t*_2_], proves to be 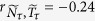. This negative value is in harmony with the expectation that the NAO signal and the Mediterranean temperature are anticorrelated. The problem with this result is that it is only one single value, in spite of the fact that the climate undergoes considerable changes during the observation period, which is accompanied by a time dependence of the underlying probability distribution (mostly during the ramps between years 600 and 700, and 1050 and 1150). If this is so, these probability distributions, characterizing the different kinds of climate, cannot be reflected faithfully by one single (i.e., time-independent) value.

A natural idea is to segment the time axis into shorter intervals, and to calculate the correlation coefficient for each of them. For the detrended time series of Fig. 1, the correlation coefficients obtained for the subsequent intervals [*t*_1_, *t*_2_] of length Δ*t* = 101 yr are presented in Table 1. The results indeed exhibit a strong dependence on time: during the CO_2_ plateau of 720 ppm, the correlation turns out to be considerably weaker than during the CO_2_ plateau of 360 ppm. It may be surprising that even positive values can be found.

**Figure 2:**
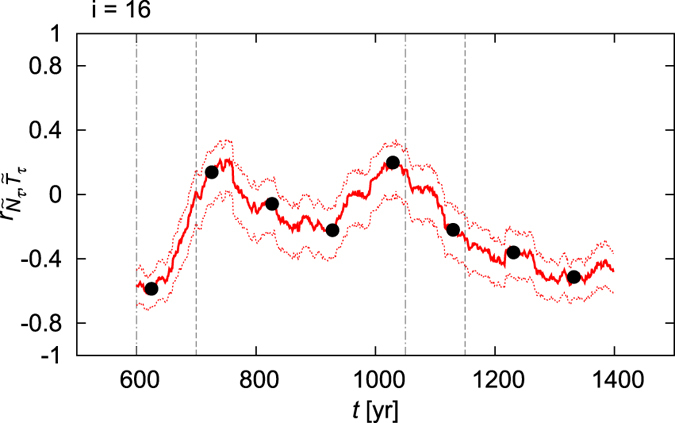
The “time evolution” (see the main text for details) of the correlation coefficient between the detrended NAO signal 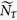 and the detrended DJF surface mean temperature 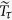 of the Mediterranean ofFig. 1, evaluated over moving time intervals of length Δ*t* = 101 yr. For each time instant *t*, the lower and the upper bound of the 95% confidence interval is given by the red dashed lines. The black circles mark the values of Table 1.

For an overview of the time dependence, we plot in red in Fig. 2 the correlation coefficient of the detrended time series of Fig. 1 with a moving window [*t*_1_, *t*_2_], of length Δ*t* = 101 yr. For each interval [*t*_1_, *t*_2_], we plot the corresponding correlation coefficient at the midpoint (at time *t* = (*t*_1_ + *t*_2_)/2) of the interval. Then, one might think of the red solid line of Fig. 2 as the “time evolution” of the correlation coefficient. This “time evolution” contains strong trends and two local maxima, characterized by *positive* correlation values, near the end and the beginning of the increasing and the decreasing ramp, respectively. One might obviously think that these maxima are related to the climate changes induced by the ramps.

Now we ask the question if these results are reliable, i.e., if they reflect the characteristics of the plethora of the parallel climate realizations at all? To give an answer, one approach is to give a confidence interval for each calculated correlation coefficient, i.e., for each time instant *t* in Fig. 2. The boundaries of the 95% confidence interval, calculated via Fisher’s *z*-transformation, are marked in Fig. 2 by red dashed lines, and this would justify at least the main trends. However, the traditional calculation of the confidence interval assumes that the underlying probability distribution is the *same* for all of the samples included in the calculation of the correlation coefficient itself, which is, due to the time evolution, not the case here.

**Figure 3:**
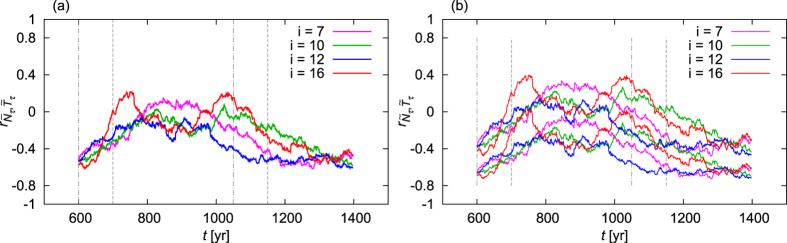
The “time evolution” (see the main text for details) of the correlation coefficient between the detrended NAO signal 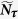 and the detrended DJF surface mean temperature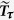 of the Mediterranean, evaluated over moving time intervals, in four different climate realizations which are marked by four different colours (see the legend). Panel (a) shows the centre of the estimate of the correlation coefficient, while panel (b) shows the 95% confidence intervals around the lines of panel (a).

To correctly illustrate how unreliable the correlation coefficients of Fig. 2 are, we sample directly the underlying probability distribution: we plot results for *further realizations* of the climate dynamics, too, i.e., for further members of the ensemble, marked in Fig. 3a by magenta, green and blue. Note that each of these lines can equally be considered as a result calculated from historically registered time series for the sea-level pressure and the temperature in our model climate. Figure 3a exhibits a striking spread among the results. One concludes that a result originating from one particular realization is practically useless. For example, even the two maxima seen in Fig. 2 turn out to be spurious consequences of random fluctuations. In Fig. 3b we consider, additionally to the lines of Fig. 3a, their corresponding 95% confidence intervals. At several *t* values, the confidence intervals are *separated*, see, in particular, the interval between *t* = 1000 yr and *t* = 1100 yr with the confidence interval of the blue, the red and the green lines. These non-overlapping confidence intervals found for the different realizations would imply statistically significant differences between these realizations! This indicates that the method for estimating the confidence intervals and hence the correlation coefficients is *not representative* from the correct point of view, i.e., from that of the parallel climate realizations.

**Figure 4:**
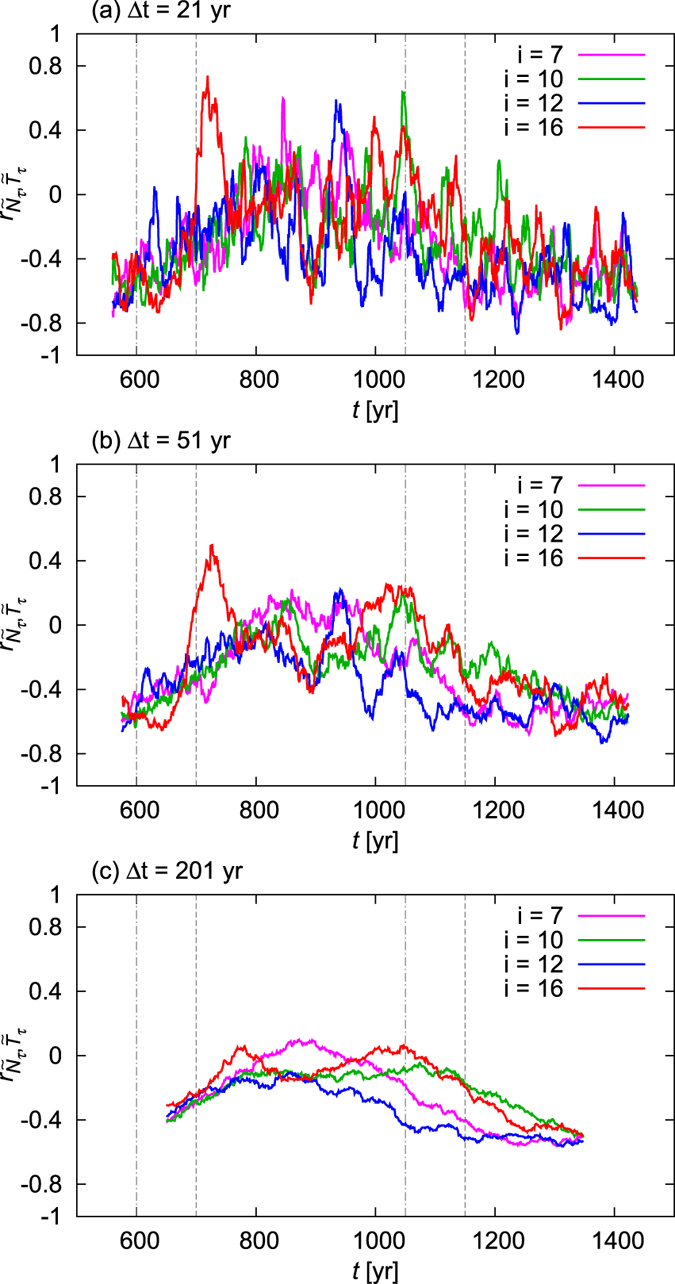
Same as Fig. 3a for different window lengths Δ*t* as indicated in the panels.

One might try to overcome this problem by choosing a more appropriate interval length Δ*t* for the calculation of the correlation coefficient. Of course, shortening the interval leads to an even stronger spread among the realizations, as illustrated by Fig. 4a,b. The other option, taking a longer interval, has an other undesired effect: it washes together different kinds of climates. In Fig. 4c Δ*t* is longer than the CO_2_ ramps themselves so that the CO_2_ plateaus of 720 ppm and 360 ppm are already washed together. Nevertheless, the spread among the different realizations is still pronounced. Increasing the interval length Δ*t* even more (not shown), in order to reduce the spread, leads eventually to washing together the entire period of observation, as discussed in the second paragraph of this section. We emphasize now that in principle *any nonzero interval length* Δ*t* is inappropriate, since the probability distribution underlying the climate dynamics is always changing in time, hence each time instant is characterized by its own correlation coefficient which should *not* be calculated from the time evolution of a trajectory over some finite time interval. The faults detailed here apply to all methods, even to the most sophisticated ones, that characterize teleconnections based on an individual realization (should this realization even be the observed history of the real Earth system).

The only way out of this situation is, instead of calculating the correlation coefficients over time, to sample the underlying probability distribution directly in each time instant, taking the samples from the *ensemble* of the possible realizations permitted by the climate dynamics. For a more detailed analysis of the difference between temporal and ensemble averages, i.e., of the breakdown of ergodicity during climate changes, see ref. 42.

### New approach: Calculating statistics over the ensemble of parallel climate realizations

The concept of parallel climate realizations enables one to define a new, ensemble-based averaging in each time instant. This is *free* of any subjective choice (e.g. of *τ* and Δ*t*) in the time-based algorithms: it can be considered to be an instantaneous method. What is even more important, it naturally treats all possible realizations of the dynamics by incorporating them into the correct statistical description.

We first show that the most appropriate definition of anomalies is provided by the framework of parallel climate realizations. To obtain the ensemble-based anomaly *A* of the NAO signal, we take the average and the standard deviation over all the parallel climate realizations, i.e., the members of the ensemble. After this, we calculate the difference of the NAO signal, *N*, and its ensemble average, divided by the ensemble standard deviation:


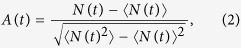


where 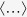 denotes averaging with respect to the ensemble. This is performed in any “instant of time”. (By an instant of time, we mean here the winter season of a year. This is appropriate, because a season is much shorter than the characteristic time scale of the climate change.)

**Figure 5:**
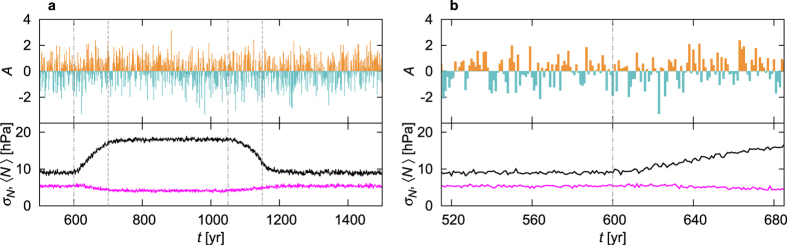
The ensemble-based anomaly *A* (2) of the NAO signal as a function of time in a single realization (*i* = 7). Panel (b) is a blow-up of panel (a). The vertical dot-dashed (dashed) lines in grey mark the beginning (end) of the CO_2_ ramps in both panels. For comparison, the ensemble average 〈*N*〉 (in black) and the ensemble standard deviation *σ*_*N*_ (in magenta) are also displayed. It is the standard deviation *σ*_*N*_ which represents the size of the characteristic fluctuations about the ensemble average, i.e., the internal variability of the NAO signal. This quantity is observed to not change dramatically during the climate change of our model.

Figure 5a shows an example for the time series of the anomaly *A*, determined in each instant of time by subtracting from the NAO signal (1) its instantaneous ensemble average (shown in black as a function of time in the same figure) and then dividing by its ensemble standard deviation (shown in magenta in the same figure). (We note that a convergence of the ensemble to the attractor was checked [see the first 200 years in Fig. 1 of ref. 15] to take place on the time scale of a century after the initiation at *t* = 0, thus in the time range shown, *t* > 500 yr, all quantities refer to those on the snapshot attractor; see discussion in the Introduction.) We emphasize that this kind of anomaly, *A*, is free from any reference to the past or the future in the form of time averages: below-than-average (negative) and above-than-average (positive) values reflect the anomaly with respect to the instantaneous climate, which we have defined as the full plethora of the ensemble in a time instant. Furthermore, this approach can also be considered as the *most appropriate detrending*. This is emphasized by Fig. 5b which is a blow-up of 5a: no trend is observed in the anomaly after the beginning of the climate change (i.e., for *t* > 600 yr). Instead, within the course of one and a half centuries, fluctuations about zero dominate the time series of the anomaly *A*.

The signature of climate change is in fact not supposed to be easily observable in anomalies evaluated with respect to the ensemble average of the instantaneous climate, since it is irrelevant whether this climate is changing in time or not. On the contrary, climate change is easily recognizable in quantities that are not detrended. In particular, climate change should be extracted from ensemble averages. The black line in Fig. 5b illustrates that the ensemble average of the original NAO signal (1) traces out clearly the sudden change of the climate (a “hockey stick”) within the same 1.5-century interval in which the anomaly is shown (and does not exhibit any trend). The fact that the averages are constant before *t* = 600 yr shows that the attractor becomes time dependent precisely by the onset of climate change.

Beyond defining anomalies, the framework of parallel climate realizations also opens the opportunity to calculate the correlation coefficients over the ensemble. In fact, these correlation coefficients are obtained, by definition, as the ensemble average of the product of the anomalies of the corresponding quantities. (As a consequence, a correlation coefficient between the anomalies is the same as that between the original quantities.) The correlation coefficients evaluated with respect to the ensemble in any time instant characterize properly the instantaneous strengths of the teleconnections. Qualitatively speaking, these correlation coefficients, taken over the ensemble of the parallel climate realizations, provide an objective characterization of the typical strength of correlations over all possibilities.

**Figure 6:**
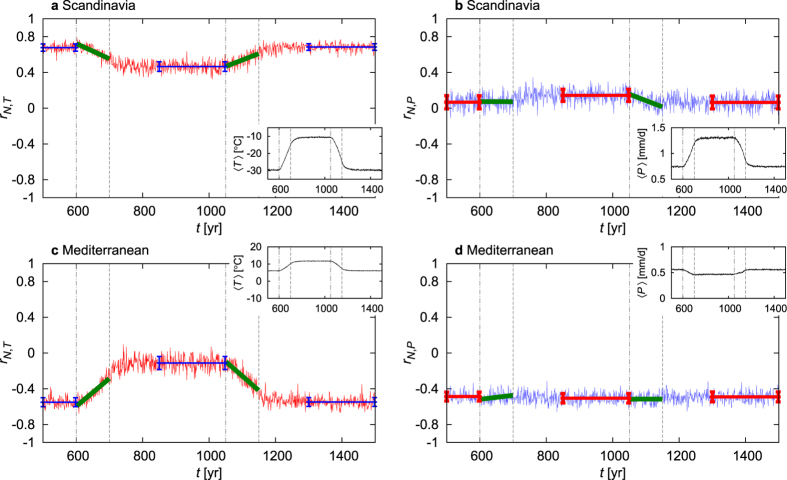
The ensemble-based correlation coefficient of the NAO signal *N* with the DJF surface mean temperature *T* (red, *r*_*N,T*_) and precipitation *P* (blue, *r*_*N,P*_) as a function of time calculated in each time instant *t* with respect to the ensemble for the geographical locations indicated in the panels. Linear fits to these time series for the intervals of the CO_2_ ramps are displayed in dark green. Temporal averages of the time series for periods of stationary values are shown in inverted colour, the error bars correspond to the temporal standard deviations on the particular time intervals. The insets present the time evolution of the ensemble average of the winter surface mean temperature 〈*T*〉 or precipitation 〈*P*〉 of the given region. The vertical dot-dashed (dashed) lines in grey mark the beginning (end) of the CO_2_ ramps in all plots.

We numerically evaluate the correlation coefficient *r*_*N,T*_ and *r*_*N,P*_ between the NAO signal (1) and the DJF surface mean temperature *T* and precipitation *P* for each year of our full scenario. Figure 6 shows the correlation coefficients for Scandinavia and for the Mediterranean (as defined in section The extraction of a NAO signal), investigated most often in the literature. One sees that the intervals of the CO_2_ ramps exhibit a kind of transition between the plateaus corresponding to constant CO_2_ concentration. This means that climate changes may yield either strengthening or weakening in the correlation coefficients. In particular, as the Mediterranean case suggests, the character of the teleconnection might change: while the correlation in one quantity (*T*) may disappear (a conclusion hardly extractable from analysing single time series, see section The ambiguity of the results based on single realizations), that in another quantity (*P*) may remain nearly intact. Note that the linear fits (marked by green) for the ramp intervals do not simply connect the values (marked by horizontal lines) corresponding to the stationary climates. In particular, the linear fits do not reach the values of the stationary climates by the ends of the CO_2_ ramps. This emphasizes the fact that the climate change itself has a delay compared to the increase or the decrease of the CO_2_ concentration (cf. Section 5 in ref. 15). This also implies that the particular time dependence during the CO_2_ ramps may not be inferred from the stationary climates. A surprisingly pronounced occurrence of this phenomenon can be seen in the case of the correlations regarding Greenland, see Supplementary Figure S6.

The amount of the overall difference in the value of the correlation coefficient between its stationary values (observable on the plateaus) depends on the geographical location and on whether the *T* or *P* correlation with *N* is investigated. In general, the correlation coefficient for the temperature is more sensitive to the climate change than the correlation coefficient for the precipitation, which is supported also by the two further geographical regions considered in Supplementary Figure S6. While in certain cases we see hardly any difference between the values of the plateaus (marked by horizontal line segments in inverted colour), in some other cases the correlation coefficient differs by a factor more than 2. This implies that the strength of correlation between climate-related patterns of geographically remote (but fixed) locations might change dramatically in a changing climate. In order to call the attention to how different these trends are from those of the variables *T* and *P*, we display in insets in Fig. 6 the ensemble average of these latter quantities.

Now that we have a solid basis on how to calculate correlation coefficients reflecting the full plethora of the parallel climate realizations, it is worth revisiting the definition (1) of the NAO signal itself from an ensemble point of view, and recalculating the correlation coefficients. This details and the results of this investigation are given in Supplementary Discussion II.

## Discussion

One important message of our study, a hint on which was given motivated by Fig. 1 and is discussed in detail in Supplementary Discussion I, is that any trend related to externally induced climate changes appearing either in any undetrended NAO signal or in the mean of any anomaly (this is possible only for anomalies evaluated with respect to some time window) should not be interpreted as a shift toward a particular sign (a particular “phase”) of the North Atlantic Oscillation and of the corresponding teleconnection pattern, in contrary to what appears in some works, e.g. refs 25,43, 44, 45, 46. This conclusion already follows from results in the literature concerning correlation coefficients evaluated over single time series in other areas and broader topics^29^,^30^,^31^.

Correlation coefficients characterize the strengths of teleconnections. We have demonstrated that the traditional evaluation of correlation coefficients over *time*^26^,^27^ depends on the length of the time intervals *τ* and Δ*t* used for detrending and for the evaluation of the correlation coefficients themselves respectively, but even more on the particular realization. An appropriate detrending is only achievable if one can completely separate internal variability from the response of the system to the external forcing (e.g. increasing CO_2_ concentration). This separation can be, in fact, carried out^7^,^15^ in the snapshot framework, i.e., in the framework of parallel climate realizations, so that an appropriate detrending, of any single time series, would be achievable. However, the framework of parallel climate realizations offers, instead of this, a superior option: it allows also a direct evaluation of correlation coefficients at each particular time instant, by taking all averages over the *ensemble*. Within this framework, above-than-average and below-than average values are naturally defined with respect to the ensemble average. Since averaging over the ensemble can be carried out in any instant of time, this framework opens the opportunity to study the time evolution of correlation coefficients and, in a wider sense, that of teleconnection patterns.

Due to the outstanding capability of the framework of parallel climate realizations that it separates externally induced trends from internal fluctuations, and since this method is mathematically totally supported and is devoid of any subjectivity, we do believe that this, i.e., the use of an ensemble after taking care of its convergence to a dynamical snapshot attractor, should be incorporated into the analysis of climate projections obtained in any large-scale climate model.

## Methods

### The snapshot approach

The concept of snapshot attractors was introduced in the physics literature to handle the dynamics of arbitrarily forced systems^6^,^7^. From a mathematical point of view, they are the attractors of nonautonomous dissipative dynamical systems and are also called pullback attractors^1^,^8^,^9^. They first appeared in a climatic context by the latter name^1^,^4^,^5^,^9^,^47^. Qualitatively speaking, a snapshot or pullback attractor appears in a system with an arbitrary, non-periodic forcing, and it is a unique object of the phase space, to which an ensemble of trajectories converges within a basin of attraction. As a consequence of the dissipative nature of the dynamics, the initial condition of the ensemble is “forgotten”: after some time, the evolution of the particular ensemble becomes *independent* of its initial position within a basin of attraction. The attractor characterizes thus the long-term behaviour of the initially different trajectories, along with the distribution traced out by the ensemble, a probability distribution which is well-defined and *unique*^7^,^15^. The snapshot attractor and its distribution may, of course, depend on time, and this is uniquely determined by the forcing scenario of the system. We consider the results of an ensemble simulation to describe *parallel climate realizations* after the initial conditions are “forgotten”, i.e., when the ensemble members reflect the dynamics on the snapshot attractor and correspond to different possible time evolutions within the same climate scenario (irrespective of the choice for the initialization of the ensemble).

The snapshot approach can be contrasted with the traditional one dealing with 30-year or multi-decadal temporal averages along single realizations. In contrast to the latter averages, the snapshot attractor and the corresponding averages, taken over the ensemble, are always related to instantaneous properties. This means that even if the climate is changing, the ensemble average is able to give a relevant statistics in any instant of time. On the contrary, temporal averages in any nonperiodically forced system always differ from the ensemble average^7^,^15^, dynamics related to climate changes are thus unavoidably nonergodic^42^.

### The Planet Simulator and the model scenario

In the present study we use mainly the default PlaSim setup with the lowest resolution (which is T21, corresponding to a roughly 5.6° × 5.6° grid of Gaussian type). The atmosphere is coupled to a non-moving mixed layer ocean^48^ without any hydrodynamical activity. The atmospheric dynamics is described by the primitive equations, which represent the conservation of momentum and mass, the first law of thermodynamics and the hydrostatic approximation. The equations are formulated for vorticity, divergence, the logarithm of the surface pressure, and temperature. They are solved via the spectral method applied to the sphere^49^. The unresolved processes are parametrised; parametrizations consist of interactive clouds^50^, of short-51 and of long-52 wave radiation. Boundary layer fluxes of latent and sensible heat, as well as vertical and horizontal diffusion, are taken into account. Water vapour transport is treated with special care via moist air convection and parameterized large scale precipitation. The annual cycle of insolation is built in. Greenhouse gases, namely water vapour, carbon dioxide and ozone, are also parameterized, and their effect is included in the calculation of the transmissivities^52^. The global concentration of CO_2_ directly influences the radiative transfer of the atmosphere. There are no sinks or sources (as there is no absorption of CO_2_ into water and the standard configuration does not treat the biosphere), instead the CO_2_ concentration can be externally controlled even as a time-dependent forcing. A detailed description of the PlaSim model can be found in ref. 53. The only exception to the default setup is the mixed layer depth which we choose to be 200 m in order to be closer to oceanographic data and to achieve more realistic atmospheric relaxation times than with the standard PlaSim setting of 50 m. The model setup is then exactly the same as in ref. 15.

We run PlaSim with the built-in initial temperature profiles and the built-in hydrostatic atmosphere initially at rest, but we slightly perturb the initial surface pressure field in 192 different replicas. The difference among these pressure fields is a random perturbation of maximum 10 hPa. A 192-member ensemble is created this way, and each member’s time evolution is monitored from the time instant *t*_0_ = 0 of the initialization over the full observational period of 1500yr with the CO_2_ forcing scenario described below. The convergence of the ensemble to the attractor is complete by about *t* = 150 yr (see ref. 15).

The forcing, just like in ref. 15, is due to a CO_2_ scenario that contains a CO_2_ doubling, augmented, after some time, with a symmetric decrease of the CO_2_ concentration back to the original level. This reference level is 360 ppm, which is first doubled over a ramp of one hundred years at a constant rate, and, after a 350-year plateau, the concentration decreases back to its initial value, linearly, over one hundred years again. Before (after) the increasing (decreasing) CO_2_ ramp, there are plateaus in the CO_2_ concentration with the reference value, i.e., 360 ppm. The precise form of the concentration dependence is


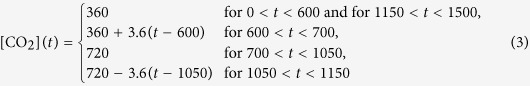


where units are year and ppm for time and concentration, respectively. The length of the CO_2_ plateaus is chosen such that a convergence (lasting about 150 years) to a stationary climate can take place much before the end of the plateaus.

### The extraction of a NAO signal

We decided to use, in harmony with the traditional definitions for the NAO index, fixed grid points for representing the NAO centres of action. We selected them such that they yield (as a few other neighbouring gridpoints, too) the usual correlations in the stationary climate with 360 ppm and, furthermore, do not fall far from the corresponding pressure extremes. The particular grid indices chosen are the following: *I*: index 60 (i.e., 28°W) in longitude and index 6 (i.e., 58°N) in latitude, 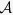: index 60 (i.e., 28°W) in longitude and index 11 (i.e., 30°N) in latitude. We take the difference in the sea-level pressure of these grid points in eq. (1) to obtain our NAO signal.

In our paper we investigate the relation of this NAO signal with surface mean quantities of Scandinavia, of the Mediterranean, of Greenland and of eastern North America. In PlaSim, we define these regions by the geographical longitude *λ* and the geographical latitude *φ* as *λ*_min_ < *λ* < *λ*_max_ and *φ*_min_ < *φ* < *φ*_max_, respectively, where the bounding values *λ*_min_, *λ*_max_, *φ*_min_ and *φ*_max_ are as in Table 2.

## Additional Information

**How to cite this article:** Herein, M. *et al*. The theory of parallel climate realizations as a new framework for teleconnection analysis. *Sci. Rep.* **7**, 44529; doi: 10.1038/srep44529 (2017).

**Publisher's note:** Springer Nature remains neutral with regard to jurisdictional claims in published maps and institutional affiliations.

## Supplementary Material

Supplementary Information

## Figures and Tables

**Table 1:** The correlation coefficient between the detrended NAO signal 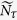 and the detrended DJF surface mean temperature 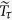 of the Mediterranean of Fig. 1, evaluated over subsequent disjoint time intervals [*t*_1_, *t*_2_] of length Δ*t* = 101 yr.

*t*_1_ [yr]	*t*_2_ [yr]	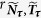	[CO_2_] [ppm]
575	675	−0.59	360…630
676	776	0.14	633.6…720
777	877	−0.06	720
878	978	−0.22	720
979	1079	0.20	720…615.6
1080	1180	−0.22	612…360
1181	1281	−0.36	360
1282	1382	−0.5	360

**Table 2:** The bounding values *λ*_min_, *λ*_max_, *φ*_min_ and *φ*_max_ for the different geographical regions considered.

Region	*λ*_min_	*λ*_max_	*φ*_min_	*φ*_max_
Scandinavia	0°	35°E	54°N	72°N
Mediterranean	10°E	36°E	30°N	44°N
Greenland	85°W	50°W	54°N	72°N
Eastern North America	85°W	70°W	27°N	45°N

See text for details.
