# Supplementary material for: The theory of parallel climate realizations as a new framework for teleconnection analysis
Source: Sci Rep. 2017 Mar 23;7:44529. doi: 10.1038/srep44529 (PMC5363062; doi:10.1038/srep44529)
Supplement: Supplementary Information [file srep44529-s1.pdf]

Supplementary Information for:

The theory of parallel climate realizations as a new framework for teleconnection analysis

Mátyás Herein[1], Gábor Drótos*[2], Tímea Haszpra[3], János Márfy[4], Tamás Tél[5]

March 6, 2017

[1]MTA–ELTE Theoretical Physics Research Group, Budapest, Hungary, and Institute for Theoretical Physics, Eötvös University, Budapest, Hungary
[2]MTA–ELTE Theoretical Physics Research Group, Budapest, Hungary, and Institute for Theoretical Physics, Eötvös University, Budapest, Hungary
*drotos@general.elte.hu
[3]MTA–ELTE Theoretical Physics Research Group, Budapest, Hungary, and Institute for Theoretical Physics, Eötvös University, Budapest, Hungary
[4]MTA–ELTE Theoretical Physics Research Group, Budapest, Hungary, and Institute for Theoretical Physics, Eötvös University, Budapest, Hungary
[5]MTA–ELTE Theoretical Physics Research Group, Budapest, Hungary, and Institute for Theoretical Physics, Eötvös University, Budapest, Hungary

Supplementary Figures S1-S6

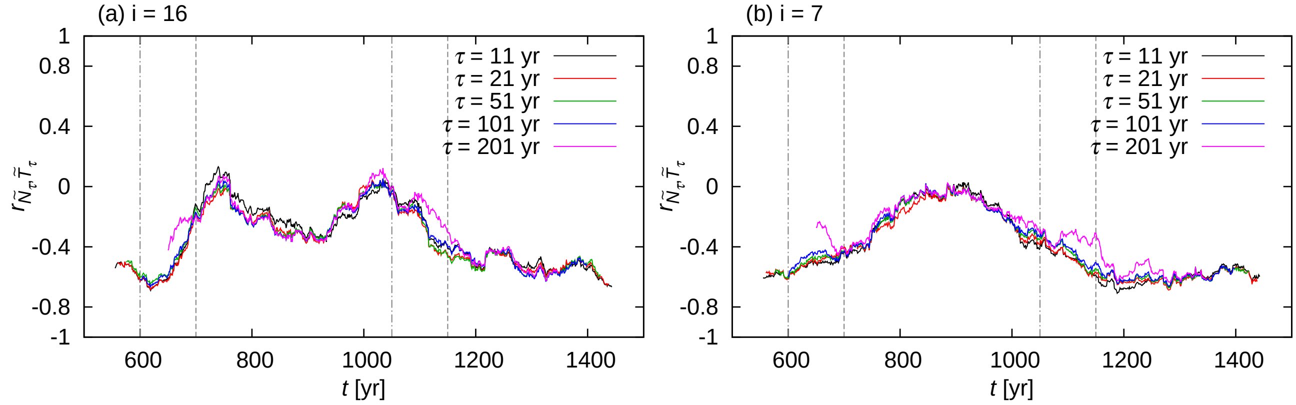

Supplementary Figure S1: The graphs of panel (a) are the same as the thick line of Fig. 2 for the canonical [25] NAO signal, but for several choices for the window length τ, as indicated in the legend. Panel (b) displays the results for a different member of the ensemble ($i = 7$). [Cf. Fig. S5 which is the same but for our NAO signal (1).]

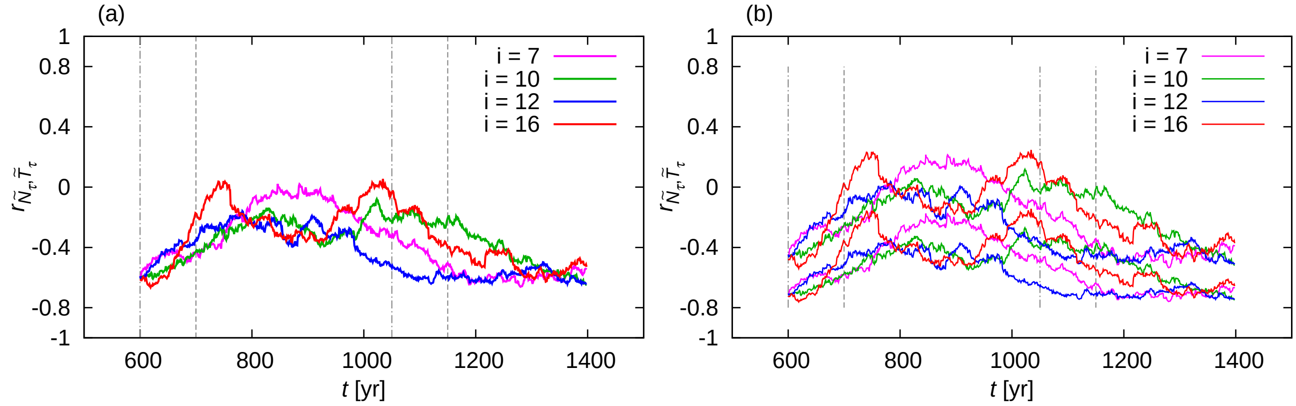

Supplementary Figure S2: Same as Fig. 3 for the canonical [25] NAO signal.

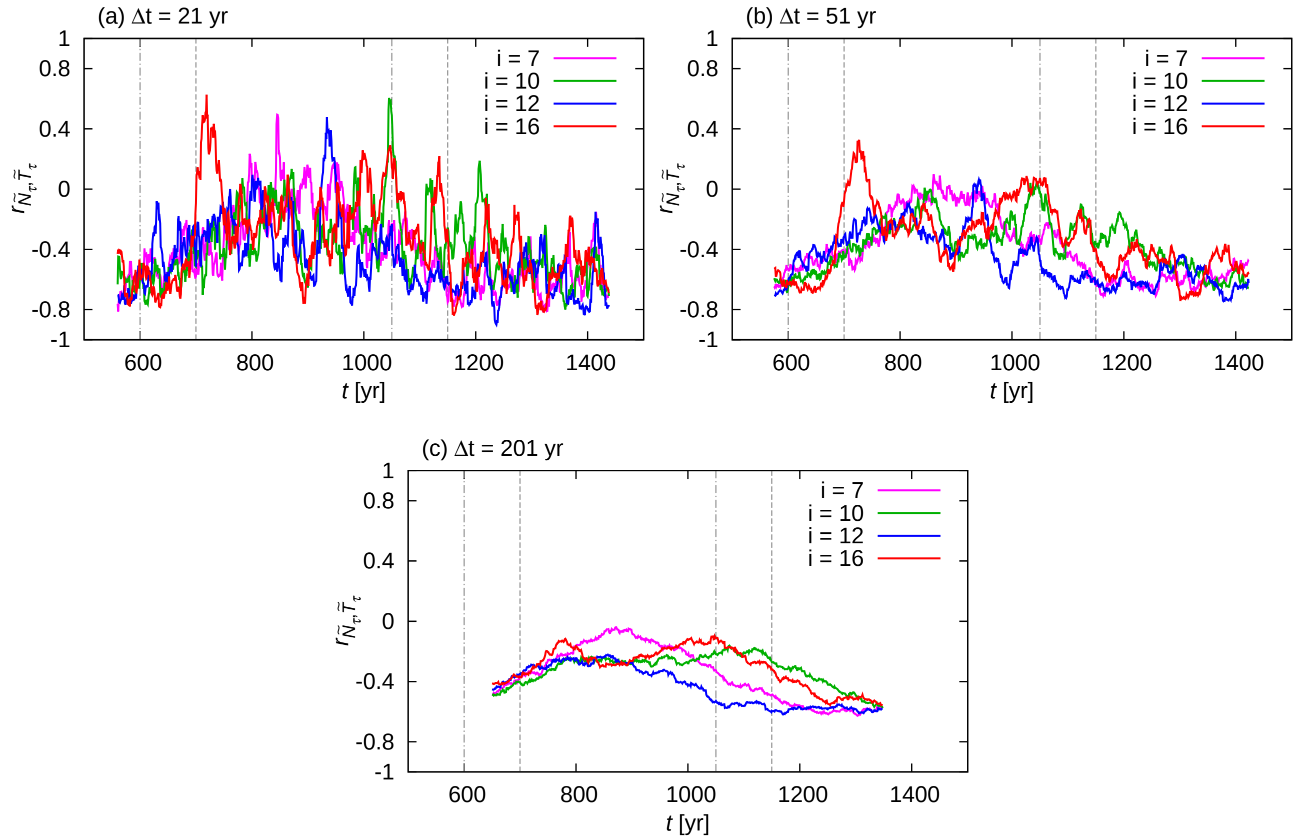

Supplementary Figure S3: Same as Fig. 4 for the canonical [25] NAO signal.

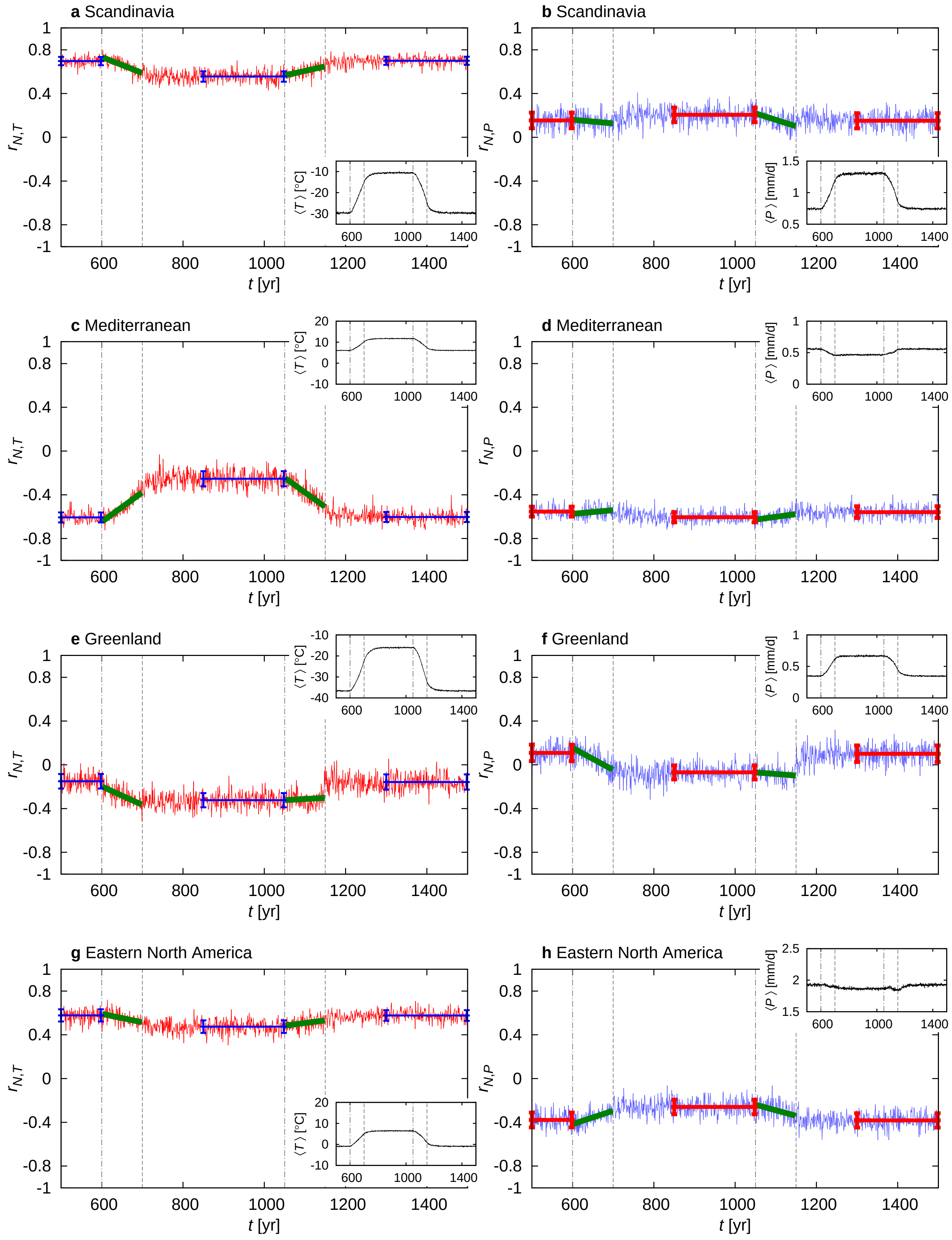

Supplementary Figure S4: Same as Figs. 6 and S6 for the canonical [25] NAO signal.

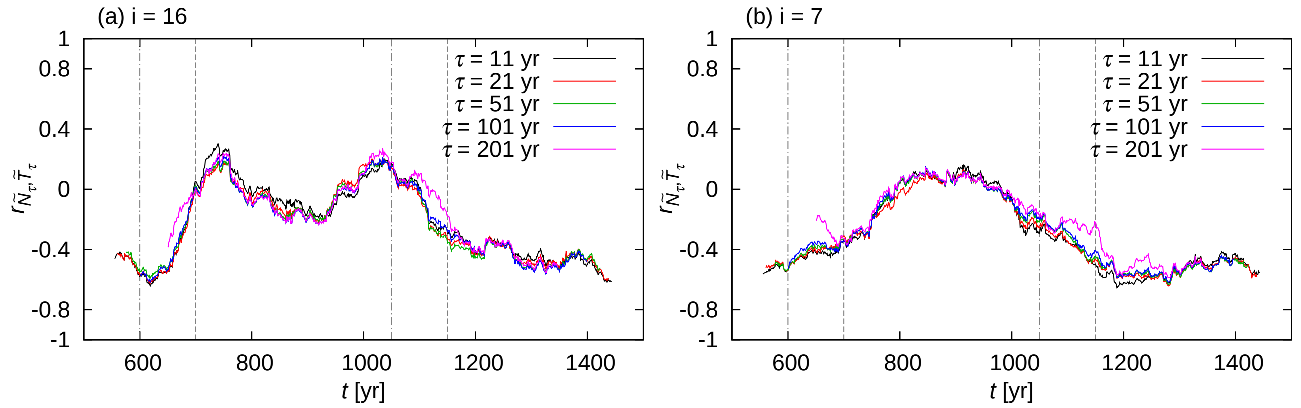

Supplementary Figure S5: The graphs of panel (a) are the same as the thick line of Fig. 2 but for several choices for the window length τ, as indicated in the legend. Panel (b) displays the results for a different member of the ensemble ($i = 7$).

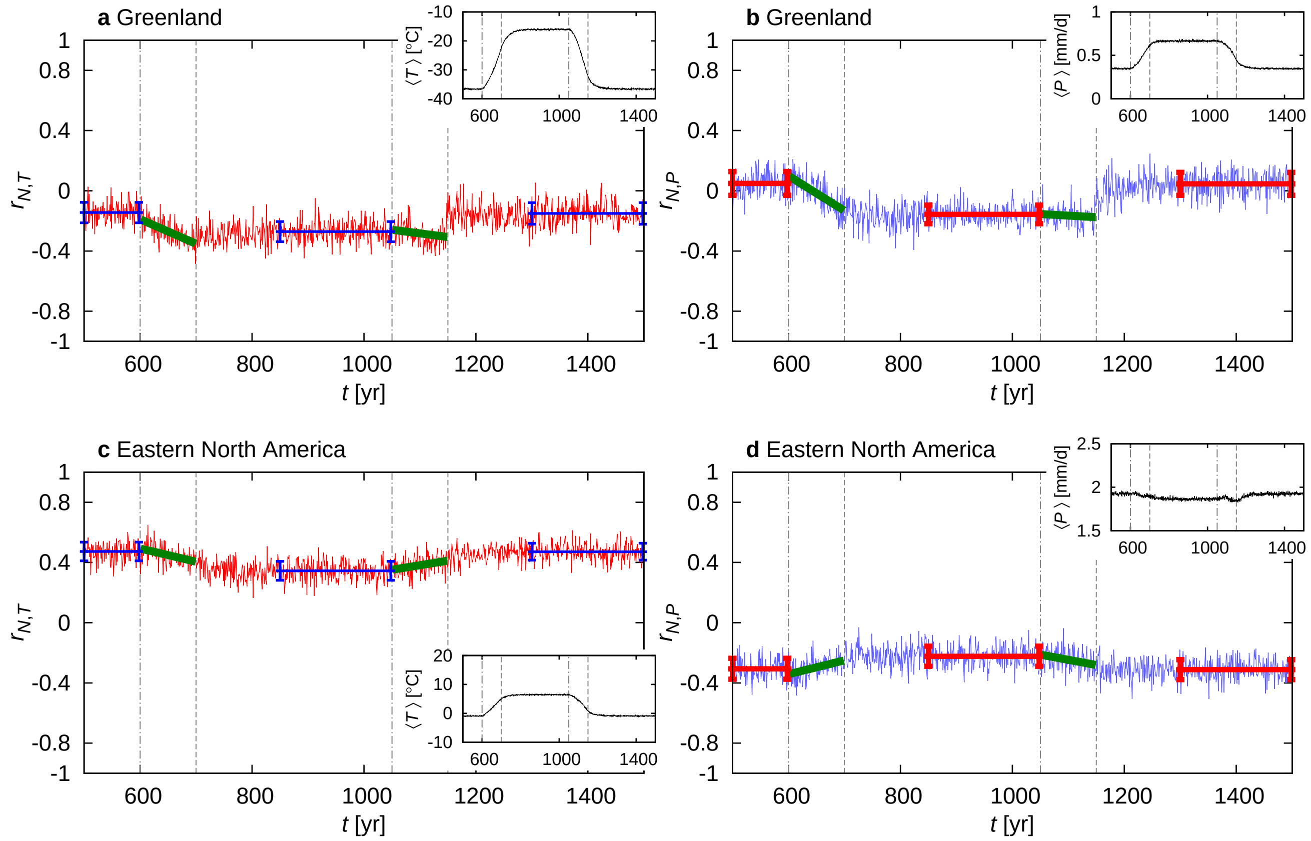

Supplementary Figure S6: Same as Fig. 6 for Greenland and for eastern North America (see subsection The extraction of a NAO signal of section Methods), as indicated in the panels.

Supplementary Discussion I: The need for detrending — an elementary illustration

One might think that the relations commonly associated with the NAO phenomenon also hold during climate changes without detrending the signals (as the methodology of some papers suggest, e.g. [25,43,44,45,46]). In order to check if this is so, let us consider Fig. S7 in which we display the NAO signal (1) and the DJF surface mean temperature of the Mediterranean: this figure is similar to Fig. 1 of the main text, but, for better visibility, the detrended time series are not included, and we took a different realization, $i = 7$, for illustrative purposes. Since an increase in the NAO signal in Fig. S7 is associated with an increase also in the Mediterranean temperature, we conclude that the naive expectation is *not* valid. Additionally, we qualitatively illustrate the positive correlation between the NAO signal and the Mediterranean temperature in a scatter plot in Fig. S8: the shape of the data set (as a whole, irrespective of the colouring) is elongated and points to the upper right. When evaluating the correlation coefficient between the NAO signal and the Mediterranean temperature with respect to time, over the full interval $[t_1, t_2] = [500, 1498]$ yr of investigation, we find $r_{N,T} = 0.50$, positive with 99.99% significance (calculated by a standard t-test), instead of a negative value.

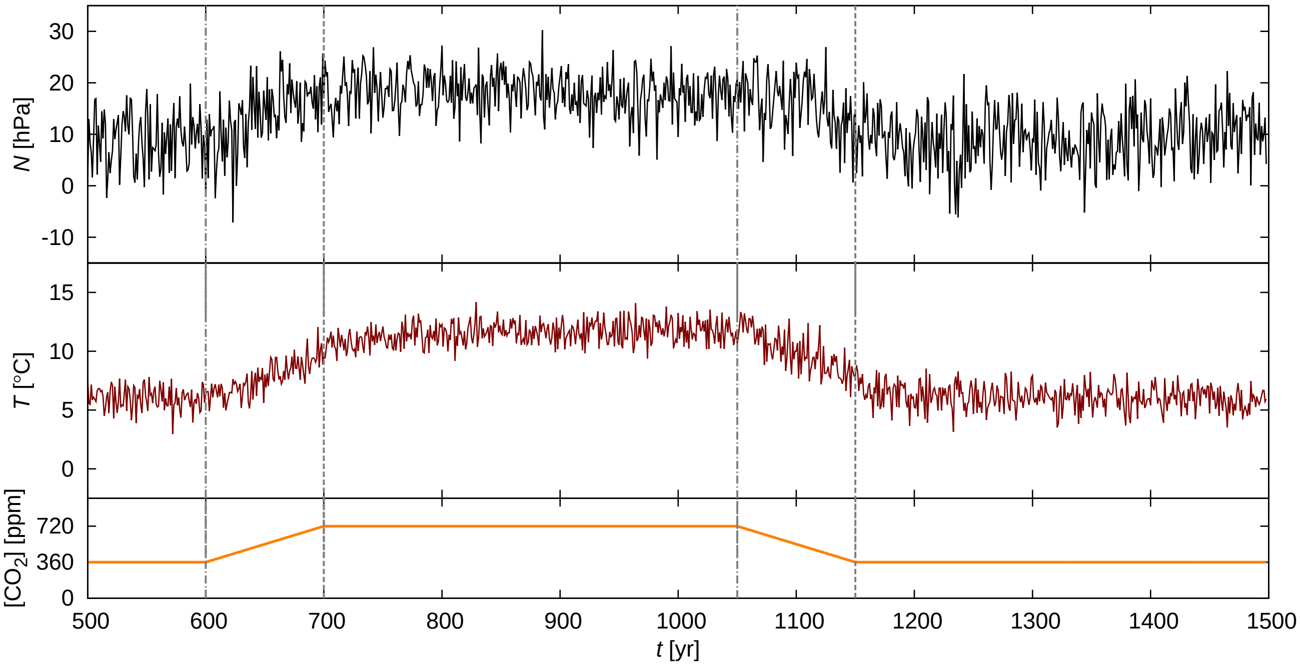

Supplementary Figure S7: The NAO signal N of (1) and the corresponding DJF surface mean temperature T of the Mediterranean in one of the particular climate realizations ($i = 7$). The forcing (i.e., the CO_2 concentration) is also plotted as a function of time. The vertical dot-dashed (dashed) lines in grey mark the beginning (end) of the CO_2 ramps.

The observed positive correlation between the NAO signal and the temperature in the Mediterranean (i.e., a high NAO signal associated with higher temperature) is due to the overall trends appearing in these quantities during climate changes. This effect is illustrated by Fig. S9a which presents the NAO signal of the investigated single climate realization as compared to its temporal average over the full interval $[t_1, t_2] = [500, 1498]$ yr of investigation. For a clear overview, we consider the anomaly of the NAO signal (denoted by $A_{[t_1,t_2]}$) defined as the value also divided by the standard deviation taken over the same time interval:

$$A_{[t_1,t_2]}(t) = \frac{N(t) - \overline{N}_{[t_1,t_2]}}{\sqrt{\overline{N^2}_{[t_1,t_2]} - \overline{N}^2_{[t_1,t_2]}}}, \tag{S1}$$

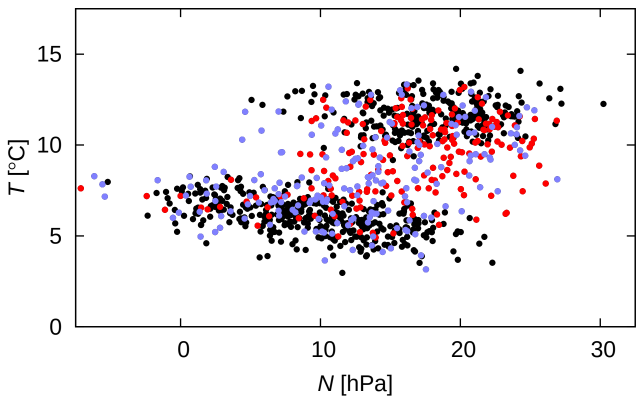

Supplementary Figure S8: The DJF surface mean temperature T of the Mediterranean as a function of the NAO signal N of (1) in one of the particular climate realizations ($i = 7$). Each dot represents a single year. Red and blue dots correspond to the climate change periods: in particular, to the time intervals of the increasing and the decreasing CO_2 ramps, respectively, with additional 90 years corresponding to the relaxation to the stationary climates. Black dots correspond to the years outside of these intervals.

where $\overline{(\ldots)}_{[t_1,t_2]}$ denotes averaging with respect to *time*, over a time window $[t_1, t_2]$. In Fig. S9a one sees that the NAO anomaly is predominantly negative in the climate with 360 ppm and positive in the climate with 720 ppm, which means that the NAO signal itself is below-than-average and above-than-average in these two kinds of stationary climate. Therefore, the values in Fig. S9a do not represent the anomaly of the NAO signal within either of these particular kinds of stationary climate. As a consequence, the commonly recognized correlation, valid within a stationary climate, may not be found (as indicated by $r_{N,T} = 0.50 > 0$).

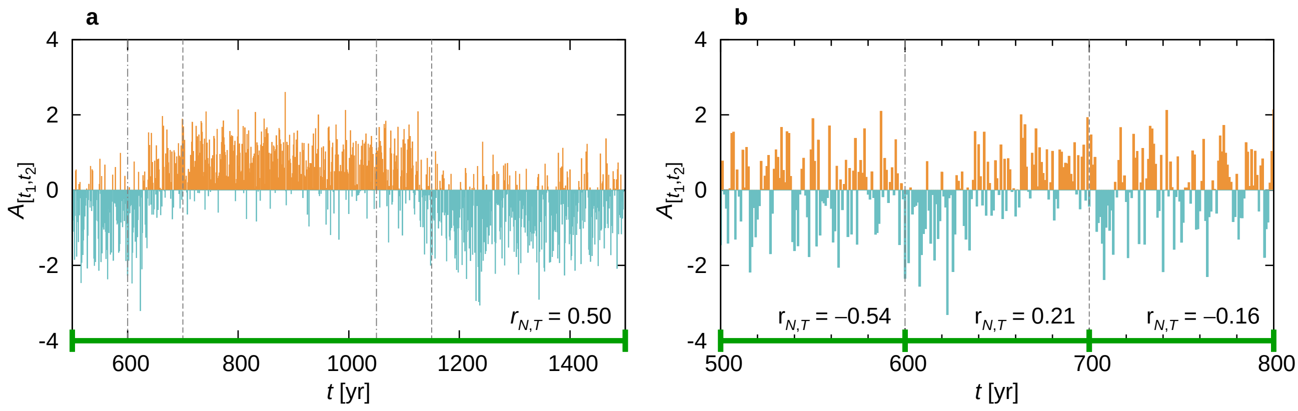

Supplementary Figure S9: The anomaly $A_{[t_1,t_2]}$ (S1) of the NAO signal as a function of time in a single realization ($i = 7$). The anomaly is calculated (a) for the full observation period $[t_1, t_2] = [500, 1498]$ yr and (b) for the subsequent, disjoint 100-year intervals, $[t_1, t_2] = [500, 599]$ yr, etc. (up to $t = 800$ yr), indicated by green on the time axis. The vertical dot-dashed (dashed) lines in grey mark the beginning (end) of the CO_2 ramps. The correlation coefficient $r_{N,T}$ between the NAO signal and the Mediterranean temperature with respect to *time*, over each interval indicated by green, is also given. The strong dependence of $A_{[t_1,t_2]}$ on the particular choice for the interval $[t_1, t_2]$ is clearly indicated by comparing e.g. year 795 in panels (a) and (b): in the former and latter, $A_{[t_1,t_2]} > 0$ and < 0, respectively.

One might try to evaluate averages and correlation coefficients over shorter time intervals than in Fig. S9a in order to characterize better the anomaly of the NAO signal. Figure S9b illustrates such an attempt with 100-year intervals. It is clear that the overall effect of the climate change still dominates

the interval $[t_1, t_2] = [600, 699]$ yr: in the first half of this interval, the anomaly $A_{[t_1,t_2]}$ is negative, and it is positive in the second half of the interval. Furthermore, the correlation coefficient $r_{N,T}$ calculated for this particular interval is positive, with 96% significance. These observations contradict the common relation between the temperature and the NAO signal (which is satisfied, however, in both neighbouring intervals — the significance for a negative correlation is 99.99% for the interval $[t_1, t_2] = [500, 599]$ yr, and it is 88% for the interval $[t_1, t_2] = [700, 799]$ yr). This suggests that during a climate change, the common correlation patterns can hold only for anomalies with respect to some signal from which the externally induced trends are removed. The observation of negative and positive anomalies in the interval $[t_1, t_2] = [600, 699]$ yr of Fig. S9b does not reflect the anomalies with respect to such a signal, since the climate properties are continually shifting in the investigated interval.

Supplementary Discussion II: The ensemble-based counterpart of the canonical [25] NAO signal and its correlation coefficients

In the main text, we define the NAO signal (1) as the simple difference in the sea-level pressure p_{sl} between the Azores and Iceland. However, the magnitude of the fluctuations in the sea-level pressure is much larger at Iceland than at the Azores, and, in order to take into account these fluctuations at the two locations with equal weight, the traditional definition of the NAO index takes the difference of *normalized* sea-level pressures [25]. Normalization is traditionally carried out by subtracting the long-term mean of the sea-level pressure and dividing by its long-term standard deviation [25], i.e., by calculating the anomaly of the sea-level pressure with respect to its long-term behaviour. In Supplementary Figures S1-S4, we demonstrate that this definition leads to qualitatively similar results to those obtained with our definition (1). We note here that defining a "long-term mean" and a "long-term standard deviation" is ambiguous in time series reflecting a changing climate, since the values of the relevant physical quantities are shifting in time [7,15].

For carrying out the normalization properly, we propose here to use the anomalies of the sea-level pressures with respect to the ensemble behaviour. In particular, we propose the following definition of the NAO signal:

$$N(t) = \frac{p_{sl,\mathcal{A}}(t) - \langle p_{sl,\mathcal{A}}(t)\rangle}{\sqrt{\langle p_{sl,\mathcal{A}}(t)^2\rangle - \langle p_{sl,\mathcal{A}}(t)\rangle^2}} - \frac{p_{sl,\mathcal{I}}(t) - \langle p_{sl,\mathcal{I}}(t)\rangle}{\sqrt{\langle p_{sl,\mathcal{I}}(t)^2\rangle - \langle p_{sl,\mathcal{I}}(t)\rangle^2}}, \qquad \text{(S2)}$$

where $\langle \ldots \rangle$ denotes averaging with respect to the ensemble. Two examples for the time series of N of (S2) are given in Fig. S10. The results for the time evolution of the investigated correlation coefficients are shown in Fig. S11. The latter results are *almost* the same as those obtained with the traditional normalization (shown in Fig. S4), but the magnitude of the numerical fluctuations is slightly smaller, which may be an indication for the proper nature of our definition (S2) for the NAO signal. In general, however, both the canonical normalization [25] and its ensemble-based counterpart enhance the magnitude of the correlation coefficients.

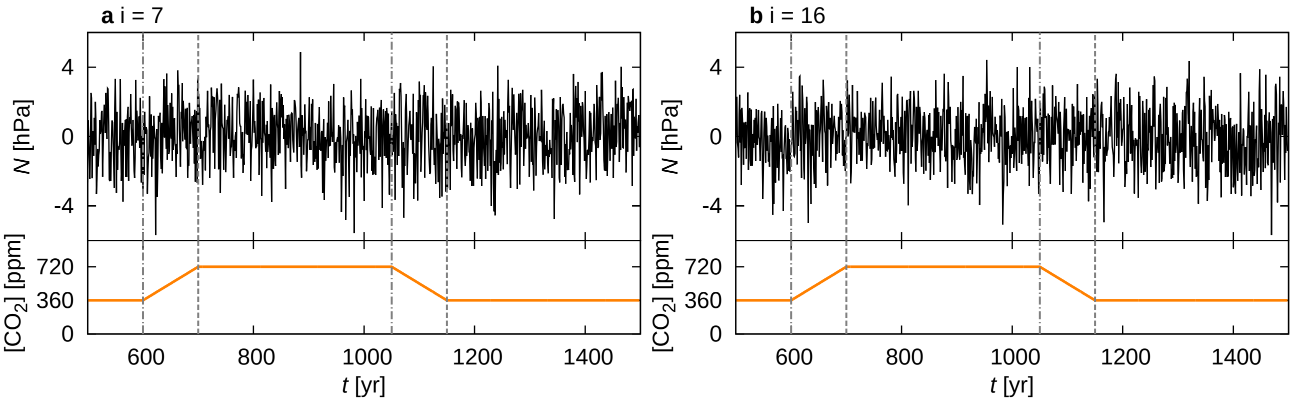

Supplementary Figure S10: The NAO signal N of (S2) as a function of time in two particular climate realizations ($i = 7$ and $i = 16$, as indicated in the panels). The forcing (i.e., the CO_2 concentration) is also plotted as a function of time. The vertical dot-dashed (dashed) lines in grey mark the beginning (end) of the CO_2 ramps.

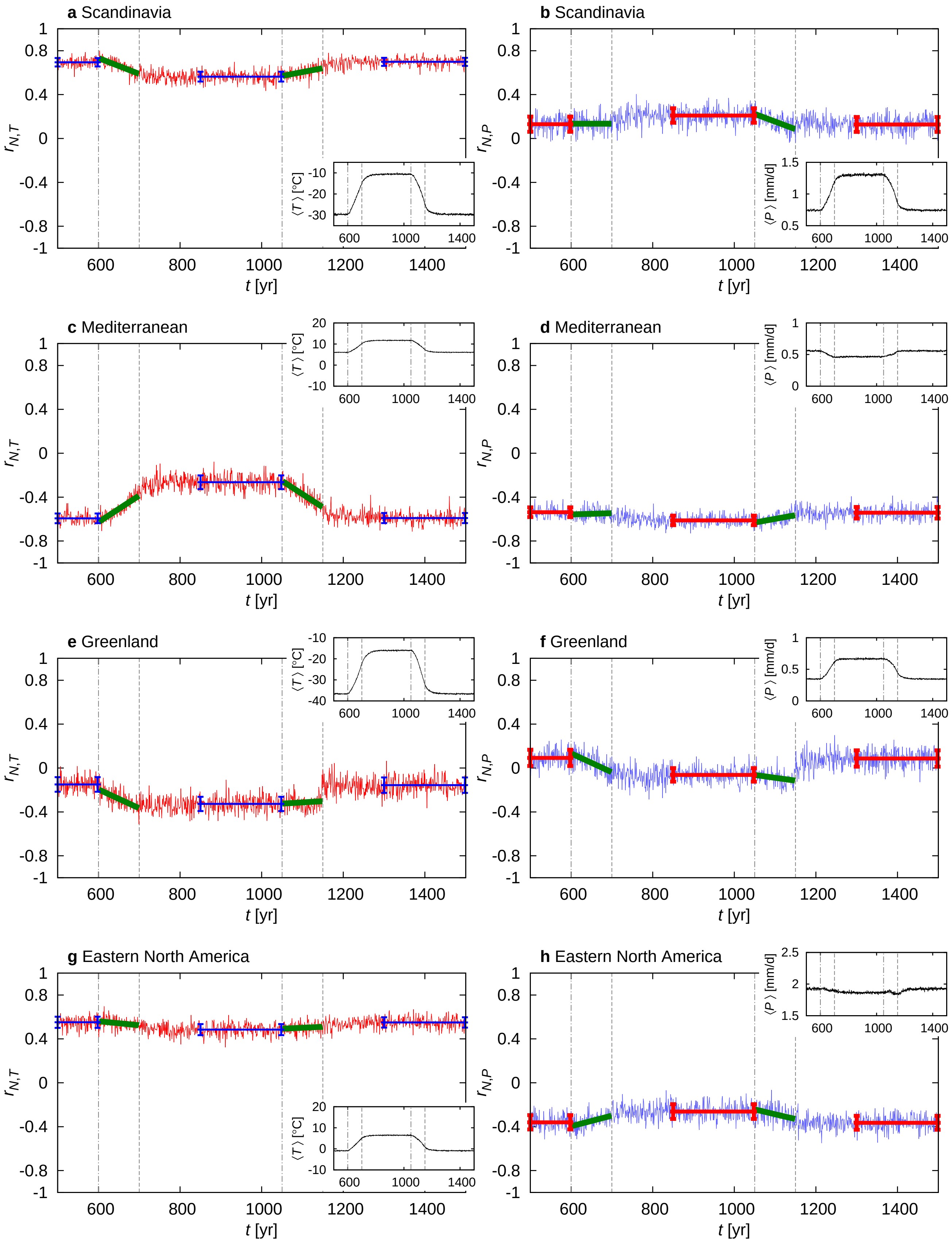

Supplementary Figure S11: Same as Figs. 6 and S6 for the ensemble-based NAO signal (S2).
